# Aim and Plausibility of Action Chains Remap Peripersonal Space

**DOI:** 10.3389/fpsyg.2019.01681

**Published:** 2019-07-17

**Authors:** Irene Senna, Lucilla Cardinali, Alessandro Farnè, Claudio Brozzoli

**Affiliations:** ^1^Integrative Multisensory Perception Action and Cognition Team (ImpAct), Lyon Neuroscience Research Center, INSERM U1028, CNRS U5292, Lyon, France; ^2^ Department of Applied Cognitive Psychology, Ulm University, Ulm, Germany; ^3^ Cognition, Motion and Neuroscience Unit, Fondazione Istituto Italiano di Tecnologia, Genoa, Italy; ^4^ University of Lyon 1, Lyon, France; ^5^ Hospices Civils de Lyon, Mouvement et Handicap & Neuro-Immersion, Lyon, France; ^6^ Center for Mind/Brain Sciences, University of Trento, Trento, Italy; ^7^ Institutionen för Neurobiologi, Vårdvetenskap och Samhälle, Aging Research Center, Karolinska Institutet, Stockholm, Sweden

**Keywords:** multisensory, grasping, peripersonal space, kinematics, motor act chains, action’s aim, naturalistic neuroscience

## Abstract

Successful interaction with objects in the peripersonal space requires that the information relative to current and upcoming positions of our body is continuously monitored and updated with respect to the location of target objects. Voluntary actions, for example, are known to induce an anticipatory remapping of the peri-hand space (PHS, i.e., the space near the acting hand) during the very early stages of the action chain: planning and initiating an object grasp increase the interference exerted by visual stimuli coming from the object on touches delivered to the grasping hand, thus allowing for hand-object position monitoring and guidance. Voluntarily grasping an object, though, is rarely performed in isolation. Grasping a candy, for example, is most typically followed by concatenated secondary action steps (bringing the candy to the mouth and swallowing it) that represent the agent’s ultimate intention (to eat the candy). However, whether and when complex action chains remap the PHS remains unknown, just as whether remapping is conditional to goal achievability (e.g., candy-mouth fit). Here we asked these questions by assessing changes in visuo-tactile interference on the acting hand while participants had to grasp an object serving as a support for an elongated candy, and bring it toward their mouth. Depending on its orientation, the candy could potentially enter the participants’ mouth (plausible goal), or not (implausible goal). We observed increased visuo-tactile interference at relatively late stages of the action chain, after the object had been grasped, and only when the action goal was plausible. These findings suggest that multisensory interactions during action execution depend upon the final aim and plausibility of complex goal-directed actions, and extend our knowledge about the role of peripersonal space in guiding goal-directed voluntary actions.

## Introduction

Recent evidence has shown that the representation of the space near the body, known as peripersonal space, might serve as a multisensory-motor interface that guides voluntary object-oriented actions such as grasping (Brozzoli et al., 2009, 2010, 2014). The existence of such representation, which relies on the integration of multisensory inputs close to the body, has been demonstrated in monkeys, as well as in humans. In monkeys, bimodal neurons in cortical and subcortical structures are activated by tactile inputs delivered to a specific body part, and by visual stimuli close to the same body part, thus promoting a body part-centered representation of the near space (e.g., Rizzolatti et al., 1981, 1988, 1997; Fogassi et al., 1992, 1996; Graziano and Gross, 1993, 1995, 1998; Graziano et al., 1994, 1997; Graziano, 1999). Behavioral and neuroimaging studies suggest the existence of homologous body part-centered representations of peripersonal space in humans (e.g., di Pellegrino et al., 1997; di Pellegrino and Frassinetti, 2000; Farnè and Làdavas, 2000; Farnè et al., 2000, 2005a,b; Pavani and Castiello, 2004; Spence et al., 2004a,b; Brozzoli et al., 2006, 2009, 2010, 2011, 2012a,b; Sereno and Huang, 2006; Makin et al., 2007; Huang et al., 2012).

It has been hypothesized that such representation plays an important role in detecting potential threats approaching the body in order to facilitate defensive reactions (e.g., Graziano et al., 2002; Cooke et al., 2003; Cooke and Graziano, 2003; Graziano and Cooke, 2006; Makin et al., 2009). Brozzoli et al. (2009) extended this view, by highlighting the role that peri-hand space (PHS) has also in guiding voluntary object-oriented manual actions. In their study, the authors used a modified version of the cross-modal congruency task, first introduced by Spence et al. (1998). In the cross-modal congruency task, participants typically hold an object between their thumb and index fingers and make speeded elevation discrimination responses to vibrotactile targets delivered to either the index finger (“up”) or thumb (“down”), while ignoring simultaneous visual distractors embedded in the object either at the same (i.e., congruent) or different (i.e., incongruent) elevation. Participants are not able to completely ignore the distractors in one modality while responding to the targets in the other modality: they are slower and less accurate when the elevation of the visual distractor is incongruent with the tactile target, a result taken to indicate the two stimuli (tactile and visual) interact. The cross-modal congruency effect (CCE, typically calculated as the difference in performance between incongruent and congruent trials on reaction times and/or accuracy) is indeed used as a measure of the interference between target tactile stimuli and visual distractors. The CCE is stronger when visual and tactile stimuli are close to each other, and thus it is used as an index of common representations of space across different sensory modalities (Spence et al., 2004a). The CCE gradually decays as the tactile stimulus and visual distractor become distant, such as when the object is moved away from the hand and outside the boundaries of the peripersonal space (see Maravita et al., 2003; Spence et al., 2004a,b). In a modified version of the original task, Brozzoli and colleagues placed the object embedding the visual distractors far from the hand, but at a reachable distance, and asked participants to discriminate tactile stimuli delivered to the hand while grasping the object (Brozzoli et al., 2009). As soon as the hand moved to reach and grasp the object, the interference between visual and tactile stimuli grew stronger (i.e., the CCE increased), as compared to a static condition, well before the hand approached the object, and the effect was specific for the acting hand. This finding shows that the execution of a simple goal-directed action triggers a dynamic on-line remapping of visuo-tactile interactions, as the action unfolds. In other words, the representation of the relative position between the tactile and visual stimuli is updated as a function of the action: the action’s target, originally distant from the hand, is “remapped” as if it were closer (i.e., inside the PPS boundaries) to the hand before it *actually* gets close to it.

A more recent study reported that such a multisensory enhancement (i.e., an increase of the CCE) starts even before the hand moves, that is, during the action planning phase (Patané et al., 2018). Thus, planning and executing voluntary object-oriented manual actions induce a remapping of the multisensory space near the hand, starting during the planning phase and continuing during early stages of action execution (i.e., at the action onset), hence well before the hand touches the object. Moreover, such anticipatory remapping of multisensory space (i.e, occurring before an actual contact between hand and object) further increases during action execution, as the hand gets closer to the target object (Brozzoli et al., 2009), and is modulated by the type and complexity of object-oriented actions, with more complex sensorimotor transformations triggering stronger visuo-tactile interactions (Brozzoli et al., 2010). These findings suggest that performing a goal-directed voluntary action, such as grasping, induces a continuous update of the spatial relationship between signals in different modalities throughout the entire action, involving sensory information near and onto the moving body part. This multisensory update might play a role in the control and guidance of the action (Brozzoli et al., 2009, 2010, 2011, 2012a,b; Makin et al., 2012; Belardinelli et al., 2018; Patané et al., 2018).

Previous studies have so far investigated this dynamic PHS remapping only in case of simple actions, restricted to one component (i.e., grasping an object) and devoid of any ecological aim. However, in the naturalistic conditions of the real world, such aimless and constrained actions are an exceptional occurrence. When we perform voluntary actions, grasping is embedded within a more complex chain of motor acts with a specific aim. Typically, we grasp an object (e.g., a candy), and then lift it to either displace it or eat it, requiring the subject to execute a series of motor steps that ultimately specify the reason why (i.e., the intention) an object has been initially reached and grasped. In these more complex actions, the goal is usually achieved only at the very end of the chain. Thus, in this case, grasping the object is not the final goal of the action (i.e., to hold the candy), but a means to an end (i.e., to eat the candy).

In the present study, we used the version of the cross-modal congruency task as modified by Brozzoli et al. (2009) to investigate visuo-tactile interactions during the execution of a relatively complex voluntary action chain, composed by an initial reach to grasp step, followed by a bring to the mouth step. The first aim was to explore the timing of PHS remapping (as measured by modulations of the CCE) during the execution of such complex action chains. As previously shown with a simpler one-step action (Brozzoli et al., 2009, 2010; Patané et al., 2018), an increase of the CCE might take place already at the beginning of the action, when the whole action is planned and started. However, according to the view that the PHS plays a role in supporting voluntary hand actions, we also anticipated that in the case of complex chains of motor acts consisting of multiple “steps,” the update of visuo-tactile interactions might be tuned to the final aim. Therefore, the multisensory effect (i.e., an increase of the CCE) might either appear in each step, or shift to a later phase of action execution, for instance taking place only once the first sub-movement toward the object is completed. In this case, to be functional to guide the hand fulfilling the main action aim (i.e., bring the object to the mouth), an updating of the visuo-tactile interactions might occur after the hand has grasped the object to be further displaced. The two alternatives are not mutually exclusive, and two moments of the action may indeed update visuo-tactile interactions: action planning (Brozzoli et al., 2009; Patané et al., 2018) and later action stages, when additional update of the multisensory information would be functional to achieve the final goal.

The second aim was to investigate whether the plausibility of an action (i.e., bringing to the mouth a piece of food that potentially could/could not enter the mouth) may affect the remapping of the PHS. Indeed, if remapping occurs when approaching the final goal of the action, it is possible to hypothesize that the plausibility of the goal achievement might induce different modulations of visuo-tactile interactions.

Finally, we aimed to explore whether two similar actions differing only in their plausibility present similar or different kinematic profiles. Previous studies reported that, when performing complex actions constituted by a sequence of motor acts, the kinematics of the initial phase is affected by the presence and the type of the subsequent one (e.g., Marteniuk et al., 1987; Gentilucci et al., 1997; Cohen and Rosenbaum, 2004; Ansuini et al., 2006, 2008; Schuboe et al., 2008; Naish et al., 2013). In other words, the kinematics of a grasping movement toward the same pen will be different whether I want to write or put the pen away. Moreover, familiarity with the to-be-grasped object influences the prior-to-contact grasping kinematics, probably because familiar objects automatically elicit the type of interactions that we habitually have with them (Gentilucci, 2002; see also De Stefani et al., 2012). In daily life, grasping a piece of food is frequently followed by the motor act of bringing it to the mouth, and it has been suggested that, at least in monkeys, viewing an eatable object may automatically activate the motor chain associated with eating (Fogassi et al., 2005). If the plausibility of the action plays a role in influencing the control of the first sub-movement, we could find differences in the kinematics of the two actions already at the initial sub-movement.

To answer all these questions, we compared two hand actions in which the same object (i.e., a cylinder) served as a support for a piece of food (i.e., a candy), and had to be grasped to bring the candy toward the (closed) mouth. Even if the two actions required the execution of the same movements, they differed in their plausibility. In one case, the candy had a horizontal orientation, compatible with the possibility for the candy to enter the mouth (plausible action); in the other, its vertical orientation made it, in principle, impossible for the candy to enter the mouth (implausible action).

Participants were instructed to reach for the cylinder with their right hand, grasp it with a precision grip, and bring the candy that was stuck on it close to the mouth (without contact), their movements’ kinematics being recorded for offline analysis. Visuo-tactile interactions were measured on-line, by having participants to decide whether they were touched on their right index finger (up) or thumb (down), while ignoring (either congruent or incongruent) visual distractors displayed on the object (Brozzoli et al., 2009, 2010).

## Materials and Methods

### Participants

Fourteen healthy naïve individuals (nine men, mean age: 20.8 ± 1.85 years), with normal or corrected-to-normal vision, took part in the study. All participants were right handed and, according to the Inserm Ethics Committee policy in terms of anonymization procedures, gave verbal consent to participate in the study, which was approved by the INSERM Ethics Committee (CEEI/IRB 00003888).

### Apparatus

**Figure 1:**
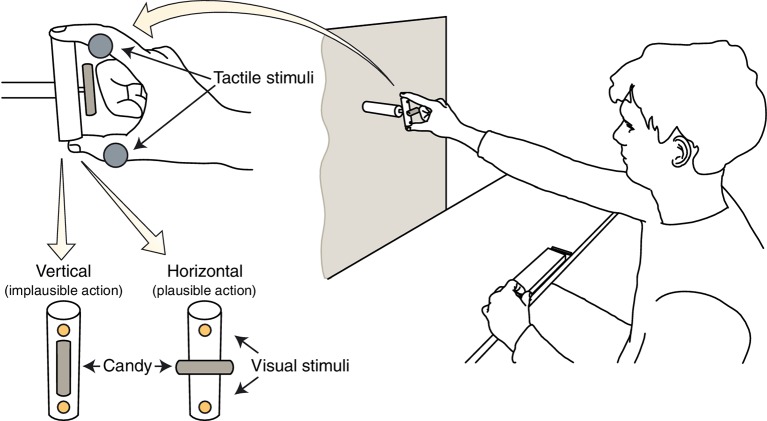
Experimental setup and task. Participants sat in front of a target object, which they had to grasp with a precision grip (i.e., with index and thumb) and bring close to the mouth. A candy was fixed on the target object, either oriented horizontally, thus potentially capable of entering the mouth (plausible action) or vertically, in an orientation that would make impossible for the candy to enter the mouth (implausible action). At the same time, they had to discriminate the position (i.e., up or down) of an electro-cutaneous stimulus (gray circles) delivered up (index finger) or down (thumb), while a concurrent task-irrelevant visual distractor (yellow circles) could be displayed on either the same (congruent) or different (incongruent) position from one of two LEDs embedded into the cylinder’s extremities.

Participants faced a vertical panel holding a wooden cylinder (7 cm height, 1.7 cm diameter), vertically aligned with their mid-sagittal plane and placed at eye level at a distance of 56 cm from the right hand starting position (see below). A 2-mm thin rod, orthogonally protruding by 6 cm from the center of the cylinder, served as a support for a candy (3.5 cm long, and without the wrapper, see Figure 1). When rotated vertically (i.e., aligned with the cylinder), the candy orientation would make it in principle impossible to enter the average participant’s max opening of the mouth (*implausible action*). Instead, when rotated horizontally, the candy size would fit the mouth opening, thus being potentially eatable by the participant (*plausible action*). Two LEDs, embedded in the cylinder at 1 cm from each extremity, were used to present visual distractor stimuli, consisting of a single flash (200 ms) delivered from either the upper or the bottom LED.

### Procedure

Participants sat at a table in front of the setup, with the thumb and the index finger of each hand laying, in a closed pinch-grip posture, on two switches fixed to the table, one on each side, and with their right foot pressing onto two pedals, one under the heel and the other under the toes. They were instructed to maintain fixation on the candy throughout the entire experimental session, and to never open the mouth. On each trial, a supra-threshold electro-cutaneous stimulus was delivered on either the index finger (up) or the thumb (down) of participant’s right hand. The stimulus was a square wave pulse (100 μs, 400 V) released by constant-current stimulators (ISO-Flex, AMPI, Israel) through self-adhesive surface electrodes (700 15-k, Neuroline, Ambu). Synchronous with the tactile stimulation onset, a visual distractor could be presented from either the upper or the bottom LED of the cylinder, thus being spatially congruent or incongruent with respect to the position of the tactile stimulation.

Participants had to judge the location of the tactile stimulus (i.e., up: index, or down: thumb) as fast as possible, while ignoring the visual distractor, by lifting the heel for thumb stimulation, and the toes for index stimulation. Visual-tactile stimulations were given at different time intervals during the motor task, consisting in two main sub-movements: (1) reaching to grasp the cylinder (i.e., the candy’s support) along its vertical axis with the index and thumb (precision grip) and (2) bringing the candy close to their mouth (without touching it). During the intertrial interval, participants repositioned the cylinder and returned their hand to the starting position, waiting for the warning sound that announced the upcoming new trial. Importantly, the cylinder was always vertically oriented, irrespective of the (vertical or horizontal) orientation of the candy, thus imposing similar movement requirements to be grasped. Participants were instructed to execute the action “as naturally as possible” with their right hand. The spatial position of the hand was recorded on line by means of an Optotrak 3,020 system (Northern Digital Inc., sampling at 100 Hz, 0.01-mm 3D resolution at 2.25 m distance). Infra-red emitting diodes (IREDs) placed on the lateral part of the nail of thumb and index finger, and on the interior part of the wrist at the styloid process level (Jeannerod, 1986), were used to record kinematics parameters.

Each trial started with an auditory warning signal followed, after a variable delay (randomized between 1,700–1900 ms), by a second auditory cue which served as a go signal for the motor task. In each trial, the visuo-tactile stimulation was randomly delivered in different phases of the two sub-movements, corresponding to one of five possible timings: (1) before movement started, randomly after 800–1,000 ms from the warning signal, and 700 ms before the go signal (*Static* condition); (2) during action planning, 200 ms after the go signal (*Planning* condition); (3) during first sub-movement execution, 200 ms after the movement onset (as determined by start switch release (*Execution* condition); (4) during object gripping (*Grasping-end* condition), when index and thumb were stationary on the object and the grip was stable (i.e., absence of any acceleration for at least 100 ms and maximum grip aperture of 7 cm ± 5 mm); and (5) during second sub-movement execution, when the object was moved (by at least 3 mm) toward the mouth (*Bringing* condition).

Overall, the experiment consisted of 16 trials (eight congruent, eight incongruent) for each of the five stimulation timings, and for each orientation of the candy (vertical or horizontal), thus yielding a total of 160 trials.

### Statistical Analyses

#### Multisensory Remapping of Peri-hand Space

Reaction times (RTs) to the tactile stimulation were log-transformed, in order to normalize their distribution, and then converted to Z-scores for each participant, to account for individual differences. As in previous studies, the (hereafter) CCE, calculated as the difference between RTs in incongruent and congruent trials, was used as an index of the amount of multisensory interference between tactile stimuli and visual distractors. A repeated measures analysis of variance (ANOVA) with the factors Candy Orientation (horizontal, vertical) and Phase (before, planning, execution, grasping-end, bringing) was conducted on the CCE scores.

#### Grasping Kinematics

The following parameters (as defined in previous work by Brozzoli et al., 2009) were analyzed: acceleration, velocity, deceleration peaks, and their relative latencies since movement onset, movement reaction times (i.e., the temporal delay between the go signal and motion onset), and movement duration (from the beginning of hand movement up to the moment in which the object has reached the minimal distance from the mouth, before being moved back to its support). For each parameter, we ran an ANOVA with the factors Candy Orientation (horizontal vs. vertical), Phase (static, planning, execution, grasping-end, bringing), and Stimulation (congruent vs. incongruent).

## Results

### Multisensory Remapping of Peri-hand Space

**Figure 2:**
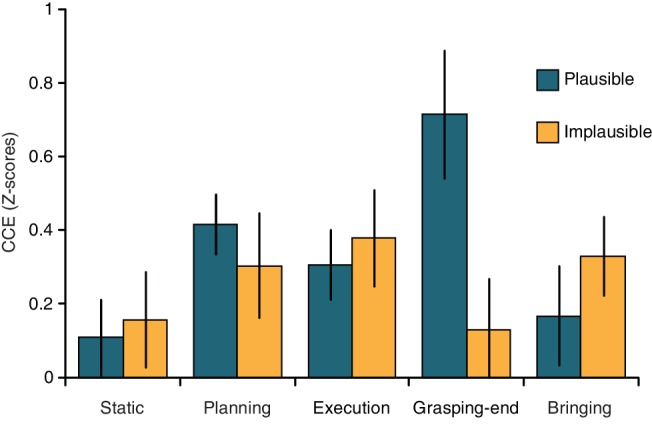
Modulation of visuo-tactile processing during action execution. Bar plots (with SEM) show the modulation of the cross-modal congruency effect (CCE) as a function of action phase and object orientation. The CCE significantly increased during the Grasping-end phase only when the action was plausible (i.e., when the candy was oriented horizontally).

Overall, participants were faster in responding to congruent (mean ± standard deviation = 0.01 ± 0.8) than incongruent trials (0.38 ± 0.8, *t*_13_ = 4.19, *p* = 0.001), thus replicating previous findings on the cross-modal congruency effect (CCE, see Spence et al., 2004a,b; Brozzoli et al., 2009, 2010). The ANOVA on the CCE values revealed a significant Candy Orientation by Phase interaction (*F*_4,52_ = 2.63, *p* = 0.044, *η*^2^ = 0.086). Duncan *post hoc* tests showed that the CCE was modulated by the phase of the action only when the candy was oriented horizontally, that is, when the action was plausible. In particular, for the plausible action, the CCE was significantly stronger during the Grasping-end condition (0.72 ± 0.72) as compared to the Static (0.01 ± 0.38, *p* = 0.006), Execution (0.31 ± 0.35, *p* = 0.049), and Bringing (0.17 ± 0.55, *p* = 0.01) phases of the action (Figure 2). The Grasping-end condition for the horizontal orientation differed also from the Static (0.16 ± 0.47, *p* = 0.009) and Grasping-end (0.13 ± 0.78, *p* = 0.007) phases of the action performed when the candy was oriented vertically (Figure 2). All other comparisons and main effects were not significant (all *p* > 0.05). For completeness, absolute reaction times for the different phases of the two actions are reported in Table 1.

**Table 1:** Absolute reaction times (mean ± standard errors of the mean, in ms) for the different phases of the plausible (horizontal orientation) and implausible (vertical orientation) actions.

Action phase	Plausible action	Implausible action
Static	514 ± 18.6	517 ± 21.3
Planning	541 ± 25.7	540 ± 25.5
Execution	542 ± 25.7	526 ± 26.2
Grasping end	477 ± 27.8	479 ± 27.8
Bringing	476 ± 28.2	480 ± 21.2

To assess whether our design had enough statistical power, we ran a *post hoc* power analysis, with the effect size we observed for the interaction (partial *η*^2^ = 0.17), the sample size of 14 participants, and alpha set at 0.05. The analysis revealed a power of 0.94, which is above the recommended 0.8 level (Cohen, 1988), thus showing that our study had an adequate power.

### Grasping Kinematics

**Figure 3:**
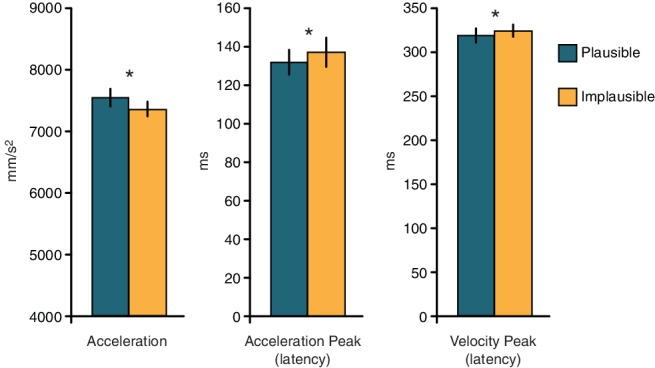
Means and standard errors of the parameters of the reaching component differing between plausible and implausible actions. Asterisks denote statistically significant differences between the actions.

The analyses on the kinematics parameters showed that kinematics was influenced by the orientation of the candy. Even if the action required to reach for and grasp its cylindrical support was the same for both candy orientations, participants showed a greater acceleration peak when the candy was oriented horizontally (7,528 mm/s^2^), than when it was oriented vertically (7,398 mm/s^2^, *F*_1,13_ = 15.38, *p* = 0.0017, *η*^2^ = 0.017). Moreover, the latency of the acceleration peak occurred earlier for the horizontal (132.5 ms) than for the vertical orientation (137.2 ms, *F*_1,13_ = 5.27, *p* = 0.039, *η*^2^ = 0.016). Similarly, the latency of the velocity peak occurred earlier for the horizontal orientation (319 ms), as compared to the vertical one (323 ms, *F*_1,13_ = 8.13, *p* = 0.014, *η*^2^ = 0.016). In other words, both acceleration and velocity peaks occurred earlier, and the acceleration peak was stronger when the action was plausible (i.e., when the candy was oriented horizontally), than when the action was implausible (see Figure 3). Movement kinematics was also partially modulated by the phase of the action at which the stimulation was delivered. The acceleration peak was more important in Planning than in any other condition, irrespective of the candy’s orientation (phase: *F*_4,52_ = 3.28, *p* = 0.018, *η*^2^ = 0.014, all *p*’s < 0.03). The analysis run on the movement reaction times revealed a significant main effect of Phase (*F*_4,52_ = 10.94, *p* < 0.001, *η*^2^ = 0.3), indicating that participants were faster in starting the action in the Static phase, as compared to any other action phases (all *p*’s < 0.001). No modulations were found in the other parameters (all *p*’s > 0.1)^1^.

## Discussion

Previous evidence has shown that, when grasping an object, the visuo-tactile interactions occurring between target object and grasping hand are updated as to remap the target location closer to the hand, well before the hand gets in contact with the object (Brozzoli et al., 2009, 2010). Such a spatial remapping of PHS, initially reported to occur at the early phases of action execution (Brozzoli et al., 2009, 2010; Belardinelli et al., 2018), has recently been shown to occur already during the motor planning phase (Patané et al., 2018). The PHS remapping continues being updated during the subsequent phases of the grasping action as the hand approaches the target, thus revealing its highly dynamic, time-sensitive nature (Patané et al., 2018). The fact that the update of the multisensory interactions leads, rather than follows, movement execution suggests that the representation of the peripersonal space might play a crucial role in guiding the execution of voluntary goal-directed actions, such as grasping (Brozzoli et al., 2009, 2010, 2012b, 2014; Makin et al., 2012).

In the present study, we assessed the modulation of visuo-tactile interactions during the execution of more complex actions, consisting of a sequence of motor acts aimed at grasping an object holding a piece of food (a candy), and bringing it to the mouth. We showed that action goal and plausibility influence PHS representation. Indeed, the visual information (on the object) interacts with the tactile stimuli (on the hand) more strongly when the hand has already reached the object (as compared to the previous action phases). Crucially, the effect is present only when the action is plausible, i.e., when the candy is oriented horizontally, thus making the action goal potentially achievable in the subsequent motor component of the action (bring-to-the-mouth). Previous research has shown that the multisensory interference is maximal when the participant is holding the object containing the visual distractors (and thus when the tactile and visual stimuli are spatially adjacent) (e.g., Spence et al., 2004a). Here, multisensory interference upon grasping increased only when the action was plausible, thus ruling out the possibility that the effect we report is driven by the mere spatial proximity between the two stimuli. In fact, were this the case, the increase in multisensory interference observed while the participant is holding the object should take place for both conditions (i.e., plausible and implausible). Instead, it was observed selectively in the plausible condition. Yet, we cannot exclude that other variables might have partially contributed to the observed effect. Indeed, while the to-be-grasped support for the candy was always vertically oriented (so that the movement required to grasp it would be comparable between the plausible and implausible actions), the orientation of the candy could be either parallel (implausible action) or perpendicular (plausible action) to the support. It is thus possible that different orientations of the candy with respect to its support may introduce perceptual differences between the two conditions. Overall, given that the CCE was comparable between the two conditions in most action phases, potential perceptual differences between the two conditions do not seem to affect task performance.

These findings suggest that the modulation of visuo-tactile interactions triggered by action planning may vary in time depending upon the final aim and plausibility of the action itself. Here, visuo-tactile interactions were not updated at early phases of the action, as it happens with simpler grasping actions (Brozzoli et al., 2009, 2010; Patané et al., 2018). Instead, such modulation occurs later in time, when the hand holds the object and is about to initiate the second action step. Moreover, movement kinematics was partially affected by the plausibility of the action. Both acceleration and velocity peaks occurred earlier, and the acceleration peak was greater when the candy was oriented horizontally (plausible action), than when it was oriented vertically (implausible action). In a sequence of motor acts aimed at reaching for an object to manipulate it, the aim of the motor steps following the grasping phase affects the kinematics of the initial stages of the action (e.g., Marteniuk et al., 1987; Gentilucci et al., 1997; Cohen and Rosenbaum, 2004; Ansuini et al., 2006, 2008; Schuboe et al., 2008; Naish et al., 2013). In particular, reaching movements are generally slower when the post-grasp movements require greater precision (e.g., Ansuini et al., 2006, 2008; Schuboe et al., 2008; Naish et al., 2013; Quinlan and Culham, 2015). For instance, an earlier acceleration peak takes place in the reaching phase of a reach-to-grasp action when the grasped object is then brought close to the mouth as compared to when it is placed in another location (Naish et al., 2013). Such differences in the kinematics reflect the fact that the action of bringing an object to the mouth requires greater accuracy and precision than the action of placing it somewhere else. Our study confirms and extends these findings, by suggesting that the prior-to-contact grasping kinematics is affected not only by the goal of an action, but also by its plausibility.

In conclusion, these results expand our knowledge regarding the link between multisensory processes dedicated to encode target objects within reaching space and the sensorimotor computations required to plan and execute a complex chain of actions. Previous research had shown that voluntary object-oriented actions induce an on-line, continuous remapping of the peri-hand space, speaking in favor of a role for peri-hand space in the motor control and guidance of actions (Brozzoli et al., 2009, 2010; Belardinelli et al., 2018; Patané et al., 2018). In the present study, we extend this previous finding by showing that the remapping of the peripersonal space is driven by the final aim of the action, and by its plausibility.

## Ethics Statement

This study was carried out in accordance with the recommendations of the INSERM Ethics Committee (CEEI/IRB 00003888). According to such recommendations, all participants gave verbal consent to participate in the study, which was approved by the INSERM Ethics Committee (CEEI/IRB 00003888).

## Author Contributions

AF, CB, and IS designed the experiment. IS and LC analyzed the data. IS, LC, CB, and AF wrote the paper.

### Conflict of Interest Statement

The authors declare that the research was conducted in the absence of any commercial or financial relationships that could be construed as a potential conflict of interest.

## Footnotes

^1^In our experiment, participants were asked to perform two tasks (i.e., perceptual and motor) at the same time. To additionally explore the effect of performing a second motor task on participants’ overall reaction times (RTs) in the different phases of the action, we ran an ANOVA on the absolute RTs (log-transformed to normalize their distribution) with the within-subject factors Phase and Candy orientation. Results revealed a significant main effect of phase (F_4,56_ = 11.67, *p* < 0.01), and *post hoc* test showed that RTs were significantly slower in the planning and execution phases as compared to the grasping-end and bringing phases (all *p*’s < 0.02). Thus, planning and starting an action overall slowed down the RTs to the perceptual task. However, faster RTs in the grasping-end condition cannot explain the effect we found in the CCE. First of all, although RTs were faster also in the bringing phase as compared to planning and execution, we do not see a modulation of the CCE in the bringing phase analogous to that found in the grasping-end phase, despite the fact that also in this phase the participant is holding the object, and he is still executing the movement. Second, the effect we found on the CCE is specific for the plausible action only. Thus, if the effect we found was driven by the mere presence of a simultaneous motor task, we should have found a similar CCE modulation for both plausible and implausible actions. Similarly, if kinematics parameters were reflecting the priority trade-off between performances in the two tasks (perceptual and motor), we should have found similar effects for the plausible and implausible actions. Indeed, the two actions are equivalent in terms of required movements, and thus an effect of having a dual task rather than one should lead to analogous kinematics parameters for both actions. Instead, we found that kinematics was affected by the candy orientation only.
